# Psychological and Brain Connectivity Changes Following Trauma-Focused CBT and EMDR Treatment in Single-Episode PTSD Patients

**DOI:** 10.3389/fpsyg.2019.00129

**Published:** 2019-02-25

**Authors:** Emiliano Santarnecchi, Letizia Bossini, Giampaolo Vatti, Andrea Fagiolini, Patrizia La Porta, Giorgio Di Lorenzo, Alberto Siracusano, Simone Rossi, Alessandro Rossi

**Affiliations:** ^1^Siena Brain Investigation & Neuromodulation Lab, Neurology and Clinical Neurophysiology Section, Department of Medicine, Surgery and Neuroscience, University of Siena, Siena, Italy; ^2^Berenson-Allen Center for Noninvasive Brain Stimulation, Beth Israel Deaconess Medical Center, Harvard Medical School, Boston, MA, United States; ^3^Department of Psychiatry, University of Siena, Siena, Italy; ^4^EMDR Italy Association, Bovisio Masciago, Italy; ^5^Laboratory of Psychophysiology and Cognitive Neuroscience, Chair of Psychiatry, Department of Systems Medicine, University of Rome “Tor Vergata”, Rome, Italy; ^6^Tor Vergata University of Rome Fondazione Policlinico Tor Vergata Roma, Rome, Italy; ^7^Department of Medicine, Surgery and Neuroscience, School of Medicine, University of Siena, Siena, Italy

**Keywords:** EMDR, fMRI, PTSD, connectivity, psychotherapy, MRI, CBT

## Abstract

Among the different therapeutic alternatives for post-traumatic stress disorder (PTSD), Trauma-Focused Cognitive-Behavioral Therapy (TF-CBT) and Eye Movement Desensitization and Reprocessing (EMDR) Therapy have shown promising results in helping patients cope with PTSD symptoms. However, given the different theoretical and methodological substrate of TF-CBT and EMDR, a potentially different impact on the brain for the two interventions could be hypothesized, as well as an interaction between trauma-specific PTSD symptomatology and response to a given psychotherapy. In this study, we monitored psychological and spontaneous functional connectivity fMRI patterns in two groups of PTSD patients who suffered by the same traumatic event (i.e., natural disaster), before and after a cycle of psychotherapy sessions based on TF-CBT and EMDR. Thirty-seven (37) PTSD patients were enrolled from a larger sample of people exposed to a single, acute psychological stress (i.e., 2002 earthquake in San Giuliano di Puglia, Italy). Patients were randomly assigned to TF-CBT (*n* = 14) or EMDR (*n* = 17) psychotherapy. Clinical assessment was performed using the Clinician-Administered PTSD Scale (CAPS), the Davidson Trauma Scale (DTS) and the Work and Social Adjustment Scale (WSAS), both at baseline and after treatment. All patients underwent a fMRI data acquisition session before and after treatment, aimed at characterizing their functional connectivity (FC) profile at rest, as well as potential connectivity changes associated with the clinical impact of psychotherapy. Both EMDR and TF-CBT induced statistically significant changes in clinical scores, with no difference in the clinical impact of the two treatments. Specific changes in FC correlated with the improvement at the different clinical scores, and differently for EMDR and TF-CBT. However, a similarity in the connectivity changes associated with changes in CAPS in both groups was also observed. Specifically, changes at CAPS in the entire sample correlated with an (i) increase in connectivity between the bilateral superior medial frontal gyrus and right temporal pole, and a (ii) decrease in connectivity between left cuneus and left temporal pole. Results point to a similar, beneficial psychological impact of EMDR and TF-CBT for treatment of natural-disaster PTSD patients. Neuroimaging data suggest a similar neurophysiological substrate for clinical improvement following EMDR and TF-CBT, involving changes affecting bilateral temporal pole connectivity.

## Introduction

Posttraumatic stress disorder (PTSD) is a psychiatric illness caused by traumatic events, usually developed after exposure to trauma such as physical or sexual assault, injury, combat-related trauma, natural disaster or death, but also after witnessing or indirect exposure (APA Association, 2013, October 3, 2013). It is estimated that, during lifetime, 60.7% of men and 51.2% of women experience at least one potentially traumatic event such as being taken hostage or being kidnaped, experiencing or witnessing sexual or physical assault, torture, a terrorist attack, a severe car accident, a natural disaster, war, or the unexpected death of a beloved person (Kessler et al., 1995). Of those experiencing potentially traumatic events, 10–40% develop psychiatric symptoms of clinical relevance (Breslau et al., 1999; Odonnell et al., 2008) such as affective disorders, substance abuse, or PTSD. PTSD is configured as a complex syndrome with pathognomonic symptomatology that includes re-experiencing of trauma-related aspects (i.e., flashbacks), avoidance of trauma-related situations, hyperarousal and emotional numbing, together with cognitive symptoms including impoverished auto-biographical memory for positive events (Harvey et al., 1998), attention and working memory deficits (Scott et al., 2015), enhanced arousal induced by trauma-related stimuli (Karl et al., 2006), as well as decreased social functioning (Fontana and Rosenheck, 2010). These features highlight the need for understanding the neurobiological basis of stress vulnerability (Brunetti et al., 2017), the impact of PTSD on the brain as well as the neural effect of treatment interventions.

Diverse pharmacological and psychotherapeutic approaches for PTSD treatment have been suggested, with psychotherapy being considered the gold standard, whereas pharmacological treatment is conceptualized as a form of symptoms control. Among the various alternatives, trauma-focused psychotherapeutic approaches such as trauma-focused cognitive behavioral therapy (TF-CBT), eye movement desensitization and reprocessing (EMDR), and exposure therapy (ET) are the most widely used (Gillies et al., 2012), with recent promising evidence also for mindfulness-based therapies (King et al., 2016a,b). Despite differences in session-to-session patient management and behavioral techniques, TF-CBT, EMDR and ET all focus on re-elaborating traumatic events or memories, favoring the emergence of new positive attitudes at the behavioral and cognitive level, leading to fear extinction and habituation. In particular, TF-CBT and EMDR further stress the cognitive component of therapeutic process, strengthening top-down cognitive control (Robertson et al., 2004). Specifically, TF-CBT helps patients to question and modify dysfunctional trauma-associated cognitions. *In vivo* or *in sensu* confrontation with trauma reminders helps patients to overcome their avoidance of trauma-related situations and thoughts, which leads to habituation and normalization of trauma memories. Besides habituation and conditioning, increased modulation of attentional processing and cognitive control are also associated to successful TF-CBT. Differently, during EMDR, patients mentally focus a trauma-associated disturbing image, memory, emotion, or cognition. As a specific feature of EMDR, the exposure is usually short and intermixed with saccadic eye movements initiated by the therapist (Herkt et al., 2014). The neurophysiological mechanism(s) behind the effect of saccadic movements is not clear, with hypotheses spanning from an unspecific, generalized relaxation achieved through activation of the parasympathetic system (followed by conditioning-based association with traumatic memories), to a decoupling between external attention and internal reprocessing of traumatic memories, which prevents patients from feeling overwhelmed (Davidson and Parker, 2001; Herkt et al., 2014).

Given the differences in treatment schedule and management, EMDR and TF-CBT could result in different therapeutic effects as well as different therapy-induced brain changes. Notably, multiple studies have addressed the impact of one or the other approach on both clinical and neurobiological patients’ profile, using neuroimaging techniques such as functional and structural magnetic resonance imaging (MRI, fMRI), single-photon emission computed tomography (SPECT), and positron emission tomography (PET) (Malejko et al. (2017). However, a direct comparison of the brain changes induced by the two interventions has not been performed. Most importantly, the type of trauma leading to PTSD has been shown to be a significant modulator of both patients’ clinical and neuroimaging profile, leading to different physical and behavioral outcomes as well as different prevalence of PTSD. For instance, natural disaster/terrorism seems more associated with cardiovascular disease, gastrointestinal disease and arthritis, while combat-related trauma is not associated with any physical condition (Husarewycz et al., 2014). As for PTSD-related brain changes, morphometric and functional brain abnormalities in PTSD patients have been shown to follow different patterns for specific types of trauma as well (Meng et al., 2016).

In the present investigation we focused on monitoring the clinical and brain impact of TF-CBT and EMDR in a sample of PTSD patients who underwent the very same traumatic experience (i.e., natural disaster, ND). We collected data on a group of PTSD patients who survived an earthquake in Italy in 2002. Patients were screened at the Department of Psychiatry of Le Scotte Hospital in Siena (Italy), and underwent both a clinical and a neuroimaging assessment based on MRI/fMRI. Patients were then assigned to a psychotherapy intervention based on either TF-CBT or EMDR. For the present study, we focused on assessing the impact of both TF-CBT and EMDR on patterns of functional connectivity (FC) as those measured via resting-state fMRI (rs-fMRI) analysis. Rs-fMRI evaluates regional spontaneous interactions that occur when a subject is not performing an explicit task, and has proved to be an informative and reliable research tool to understand individual differences in cognition (Biswal et al., 2010) as well as provide insights into the pathophysiology of neurological (Liao et al., 2010; Santarnecchi et al., 2012; Balthazar et al., 2013) and psychiatric conditions (Bassett et al., 2008; Anderson et al., 2011). Several studies have examined resting brain activity in PTSD patients (for a review see Wang et al., 2016), revealing significantly different spontaneous activity in cortical regions [e.g., superior temporal gyrus, medial prefrontal cortex (mPFC), inferior parietal lobule and middle occipital gyrus], limbic areas (e.g., the amygdala, hippocampus, insula, thalamus, and ACC), and even in the cerebellum. However, results are somehow inconsistent. For instance, some studies focusing on the insula have reported either increased (Yan et al., 2013), decreased (Yin et al., 2011; Zhu et al., 2014) or even no insula activation (Shin et al., 2009; Kim et al., 2012) in PTSD patients. As suggested above, differences in clinical profile, type of trauma and even neuroimaging analysis methods might be the cause of such variability. As for the latter, it is important to notice that several rs-fMRI studies have adopted *a priori* regions of interest (ROIs) based on theoretical models or previous reports, thus leading to inflation of positive results regarding one specific brain region or network to the detriment of a more comprehensive understanding of trauma-induced rearrangement of whole-brain connectivity. For instance, studies on the impact of PTSD on regions such as the amygdala usually report a strong support in the notion of PTSD being driven by hyper-excitability of such structure, but at the same time neglect potential changes in other structures yet to be included in models and theories (e.g., cerebellum, motor system, and thalamus). The vast majority of studies reporting amygdala-related alterations in PTSD are based on *a priori* defined ROI analysis (for a few example see Shin et al., 2005, 2009; Fonzo et al., 2010; Linnman et al., 2011; Sripada et al., 2012; Bruce et al., 2013; Stevens et al., 2013), i.e., they are explicitly looking just at the fMRI signal from the amygdala both during an emotion-provoking task or resting-state, neglecting activity in the rest of the brain. Additionally, to apply a ROI-based analysis also decrease the number of multiple comparisons and increases statistical power, resulting in a series of significant reports about one specific region that might be actually misleading for the comprehension of PTSD neurobiology.

Therefore, the present study explored the impact of EMDR and TF-CBT psychotherapy on PTSD patients’ FC patterns by adopting a validated whole-brain anatomical atlas used in previous reports (Smith et al., 2004; Makris et al., 2006), providing a parcellation of the entire brain, including cortical, subcortical and cerebellar structures. Given the theoretical and, most importantly, methodological differences between EMDR and TF-CBT, we hypothesized that (i) EMDR and TF-CBT will induce different changes in functional connectivity fMRI patterns after psychotherapy, with (ii) more pronounced changes in connectivity involving the visual system and higher-order associative regions for, respectively, EMDR and TF-CBT.

## Materials and Methods

### Study Participants and Group Assignment

In 2002, a devastating earthquake caused, among other tragedies, the collapse of an elementary school (1st–5th grade) in San Giuliano di Puglia (Campobasso, Italy). As a result, 27 children and a schoolteacher died. For the present study, 31 PTSD patients were recruited among the population affected by the earthquake, including survivors of the building collapse and victim’s family members (parents, siblings). All subjects, recruited between January and March 2012, reported a symptomatology centered around a traumatic memory related to the event. None of the subjects did undergo any previous trauma-focused psychotherapy.

Two psychotherapeutic interventions were offered to the patients, namely EMDR and TF-CBT. The patients were given the opportunity to decide when to start the therapy according to their schedules, with four treatment cycles starting between March and May 2012. Assignment to EMDR and TF-CBT was pseudo-randomized across patients, based on patients’ trauma severity at presentation. Perfect balance in severity across groups was not achieved due to the distribution of severity levels toward the third and fourth treatment cycle. The final sample of participants who completed the study (i.e., both clinical and MRI data acquired before and after psychotherapy) included 14 patients in the TF-CBT group (9 male, age = 37.7 ± 12) and 17 in the EMDR one (10 male, age = 35.4 ± 14), out of the 37 patients (83.7%) originally enrolled in the study (17/19 EMDR, 14/18 TF-CBT). Even though not significant, a difference in drop-outs for TF-CBT and EMDR was present, possibly due to the different average length of the two therapeutic interventions (10 ± 2 weeks and 4 ± 2 weeks, respectively). Please see dedicated paragraphs about each intervention for further details. Given the different protocol followed for EMDR and TF-CBT (and corresponding differences in timing of pre–post clinical and fMRI assessments), the interval between baseline and post-therapy assessments was included as a covariate in all the analyses. We did not use a fix interval for pre–post assessment and instead preferred scanning/evaluating patients right after each psychotherapy cycle, i.e., at the moment of highest probability of showing a beneficial effect on psychological dimensions and/or changes in FC patterns. The protocol was approved by the university of Siena School of Medicine institutional ethics committee. All patients were given a description of the procedures and were asked to sign a written informed consent to participate in the study in accordance with the Declaration of Helsinki. For more information about demographics and clinical information of the sample, see Table 1.

**Table 1:** Demographic and Clinical information for the TF-CBT and EMDR groups.

	TF-CBT	EMDR
*N*	14	17
Age	37.7 ± 12	35.4 ± 14
Education	12.4 ± 3	13.6 ± 4
Gender	9 M	10 M
Age at trauma	26.3 ± 9	28.6 ± 12
Previous traumatic event	39%	45%
PTSD duration	10 years	10 years
CAPS	45.7	57.6
DTS	16.6	14.1
WSAS	15.7	17.4

### Study Design

The study included a clinical evaluation and a neuroimaging data acquisition, performed before and after the cycle of psychotherapy sessions. In both occasions, patients traveled to Siena and spent 2 days performing the clinical and neuroimaging evaluations at Le Scotte Hospital. Clinical evaluations were performed by trained psychiatrists (L.B., A.F.) at the department of Psychiatry. All subjects were interviewed via the Structured Clinical Interview for DSM-IV (SCID) (First et al., 1997) and the Clinician-Administered PTSD Scale (CAPS; Blake et al., 1995), whose completion required about 2 h. All subjects were also given two self-administered psychological questionnaires, the Davidson Trauma Scale (DTS; Davidson et al., 1997) and the Work and Social Adjustment Scale (WSAS) (Mundt et al., 2002). Details about the neuro-psychiatric assessment as well as MRI data and analysis are reported in dedicated paragraphs below. The psychotherapy sessions were coordinated by one of the authors (P.LP.) and carried out by certified professionals in San Giuliano di Puglia. Psychotherapy was followed by the same clinical and neuroimaging evaluations performed in Siena.

### EMDR Therapy

The therapy followed a standard EMDR protocol (Shapiro, 2014) and was composed by eight steps. The EMDR session began with the identification of patients’ most disturbing memory of the traumatic event, as well as of any associated negative belief, disturbing emotion and its bodily location. Patients were then asked to focus on these traumatic events while following the bilateral finger movements performed by the therapist for about 30 s. After each set of horizontal movements, the patients were prompt to share any emotion/flashback/percept they have been noticing during the visual stimulation. When the patients reported no more erupting emotional burst or any other feeling related to the target memory, the therapist assessed patient’s ability to elaborate on the target with no emotional distress. The process was completed when the patient reported to be able to think about the traumatic experience with no disturbing emotions or somatic reactions. Other targets were then selected and the same procedure (i.e., trauma identification, visual stimulation, assessment) was repeated. The EMDR treatment ended when patients were able to visualize themselves in a future scenario where they were able to face the re-elaborated targets while feeling no emotional discomfort. In the present sample, the EMDR required an average of 4 weeks (±2) of weekly sessions per patient. Each session lasted for approximately an hour. EMDR was performed by two certified EMDR therapists.

### Trauma-Focused Cognitive-Behavioral Therapy (TF-CBT)

Trauma-focused cognitive behavioral therapy is an evidence-based treatment model designed to assist children, adolescents, and their families in overcoming the symptomatology resulting from the exposure to a traumatic experience (Mannarino et al., 2012). TF-CBT is a skills-based model, whose core components include, among others: Psychoeducation, Affective regulation, Cognitive processing of the trauma, Trauma narrative, *in vivo* mastery of trauma reminders, and Enhancing future safety and development. In order to allow the comparison of EMDR and TF-CBT interventions, an *ad hoc* TF-CBT protocol was implemented, following a standardized organization of between and within session procedures and targets. The first session included a narrative recollection of the traumatic event, with patients describing the event multiple times (at least two). The second session included an explanation of the therapeutic plan, relaxation exercises, trauma-focused psychoeducation and introduction to the upcoming exposure exercise. The third visit included recollection of traumatic events, exposure, and home assignments. Fourth-to-ninth visits started with (i) a review of home assignment, followed (ii) by relaxation exercises prior to exposure and (iii) psychoeducation, which included the differentiation between anxiety-based (psychological) and somatic responses to stress, strategies for managing intrusive thoughts and thoughts-blocking techniques. Tenth-to-twelfth visits included Systematic desensitization (i.e., graduate exposure therapy), with the creation of a hierarchy of stressful situation. TF-CBT required an average of 10 weekly visits (±2) to be completed in the study sample.

### Clinical Evaluation

#### Structured Clinical Interview for DSM-IV (SCID)

The Structured Clinical Interview for DSM-IV (SCID-I/SCID-II) (First et al., 1997) is a semi-structured clinical interview administered by trained clinicians and designed to yield psychiatric diagnoses consistent with DSM-IV/DSM-IV-TR (American Psychiatric Association) diagnostic criteria. The SCID is composed by open-ended questions introducing content areas, followed by a series of scripted questions. The SCID was administered via consensus of two trained psychiatrists.

#### Clinician-Administered PTSD Scale (CAPS)

The CAPS measures frequency and intensity of PTSD symptoms rated for the last-week period (Blake et al., 1995). Seventeen items describe the classical PTSD cluster symptoms: re-experiencing, avoidance and numbing, and hyperarousal. In addition to assessing the twenty DSM-5 PTSD symptoms, questions target the onset and duration of symptoms, subjective distress, impact of symptoms on social and occupational functioning, improvement in symptoms since a previous CAPS administration, overall response validity, overall PTSD severity, and specifications for the dissociative subtype (depersonalization and de-realization). The CAPS total score ranges from 0 to 136, and classifies PTSD as: 0–19: asymptomatic/few symptoms; 20–39: mild PTSD/subthreshold; 40–59: moderate PTSD/threshold; 60–79: severe PTSD symptoms; and ≥80: extreme PTSD symptoms.

#### Davidson Trauma Scale (DTS)

The DTS is a 17-item self-report measure that assesses the 17 DSM-IV symptoms of PTSD. Respondents are asked to identify the trauma that is most disturbing to them and to rate, in the past week, how much trouble they have had with each symptom. Items are rated on 5-points frequency (0 = “not at all” to 4 = “every day”) and severity scales (0 = “not at all distressing” to 4 = “extremely distressing”). The DTS can be used to make a preliminary determination about whether the symptoms meet DSM criteria for PTSD, and also provides scores for three separate subscales referring to specific symptoms related to re-experiencing, avoidance/numbing and hyperarousal. Validation work showed the DTS performed well at discriminating 67 individuals with PTSD from 62 without PTSD [area under the curve (AUC) = 0.88, *SE* = 0.02] diagnosed using a semi-structured interview (SCID; Spitzer et al., 1992).

#### Work and Social Adjustment Scale (WSAS)

The WSAS is a self-report scale of functional impairment attributable to an identified problem (Mundt et al., 2002). The WSAS is a short measure of work and social adjustment, with good validity and reliability in several patients populations (e.g., depression and anxiety) (Zahra et al., 2014). A WSAS score above 20 suggest moderately severe psychopathology. Scores between 10 and 20 are associated with significant functional impairment but less severe clinical symptomatology. Scores below 10 are usually associated with subclinical populations.

### Changes in Clinical Scores After EMDR/TF-CBT

Scores obtained at CAPS, DTS, and WSAS before and after the EMDR/TF-CBT treatments were analyzed using a repeated measures Analysis of Covariance Model (rp-ANCOVA), using a *p*-value < 0.05 and including age, gender, pre–post interval and education as covariates. Models were built for global scores as well as for each subscale of the CAPS and DTS.

### MRI Data Acquisition

The MRI data was acquired on a Philips Intera whole-body MRI scanner. Resting-state fMRI data included 178 volumes with 33 axial slices covering the whole brain, acquired via a T2 BOLD-sensitive multi-slice echo planar imaging (EPI) sequence (TR/TE = 2.5 s/32 ms; field of view = 22 cm; image matrix = 64 × 64; voxel size = 3.44 mm × 3.44 mm × 3.8 mm; flip angle = 75°). Structural imaging was performed using a whole brain T1-weighted Fast Field Echo 1 mm^3^ sequence (TR/TE = 30/4.6 ms, field of view = 250 mm, matrix 256 × 256, flip angle = 30°, slice number = 150). T2-weighted Fluid Attenuated Inverse Recovery Images (FLAIR) were also acquired to assess participants white matter integrity. Participants were provided with earplugs. Particular care was taken to minimize head motion via vacuum cushions and custom-made padding.

### fMRI Preprocessing

fMRI data preprocessing and statistical analyses were carried out using SPM8 software (Statistical Parametric Mapping^1^) and MATLAB 7.5 (the MathWorks, Natick, MA, United States). The first three volumes were discarded for each subject to allow for steady-state magnetization. EPI images were slice-time corrected using the interleaved descending acquisition criteria, and realigned and re-sliced to correct for head motion using a mean functional volume derived from the overall fMRI scans. Subject whose head motion exceeded 1.0 mm or rotation exceeded 1.0° during scanning were excluded. In order to obtain a better estimation of brain tissues maps, we implemented an optimized segmentation and normalization process using DARTEL (Diffeomorphic Anatomical Registration using Exponential Lie Algebra) (Ashburner, 2007) module for SPM8. Briefly, this approach is based on the creation of a customized anatomical template built directly from participants T1-weighted images instead of the canonical one provided with SPM (MNI template, ICBM 152, Montreal Neurological Institute). This allows a finer normalization into standard space and consequently avoids under- or overestimation of brain regions volume possibly induced by the adoption of an external template. Hidden Markov Random Field model was applied in all segmentation processes in order to remove isolated voxels. Customized tissue prior images and T1-weighted template were smoothed using an 8 mm full-width at half-maximum (FWHM) isotropic Gaussian kernel. Functional images were consequently non-linearly normalized to standard space and a voxel resampling to (isotropic) 3 mm × 3 mm × 3 mm were applied. Linear trends were removed to reduce the influence of the rising temperature of the MRI scanner and all functional volumes were band pass filtered at (0.01 Hz < *f* < 0.08 Hz) to reduce low-frequency drift. Finally, a CompCor algorithm has been applied in order to control physiological high-frequency respiratory and cardiac noise (Behzadi et al., 2007).

### Functional Connectivity Analysis

FC was calculated by computing the Pearson product-moment correlation coefficient between the average BOLD time series extracted from each brain region composing the Harvard-Oxford atlas, an anatomical atlas covering 112 cortical and subcortical structures (Smith et al., 2004). A connectivity matrix was built based on each pairwise connectivity between the 112 regions. Pre- and Post- EMDR/TF-CBT matrices were then compared using a repeated measures Analysis of Co-Variance (rp-ANCOVA) model, using a statistical threshold equal to *p* < 0.05 at the single edge (i.e., connection) level with a *p* < 0.05 False Discovery Correction (FDR) for multiple comparison. Additionally, according to the network-based statistics framework proposed by Zalesky et al. (2012), an additional threshold was applied in order to isolate regions of significant changes in connectivity not due to the intrinsic positive manifold among the entire connectivity set. Analysis was done by testing the effect of two factors, i.e., “Time” and “Treatment,” respectively, representing the data acquired before and after the psychotherapeutic interventions (2 levels = Pre, Post) and the different therapeutic approaches (2 levels = EMDR, TF-CBT). All the analyses included age, gender, pre–post interval, education and total brain volume as covariates.

In order to identify a common substrate for clinical changes observed in patients receiving EMDR and TF-CBT, patterns of overlapping changes in FC across groups were explored. In the case of regions whose connectivity profile showed similar correlations with clinical scores in both EMDR and TF-CBT groups, an additional analysis aimed at increase spatial resolution was also performed, by looking at seed-based FC changes. Specifically, selected regions were used as a seed, with their average BOLD signal being correlated with that of any other voxel of the brain, thus producing spatial correlation maps not relying on any anatomical parcellation scheme (for an example see Figure 5). For seed-based analysis, a *p* < 0.05 at single-voxel level (FDR corrected) and a *p* < 0.05 (cluster-based corrected) were applied.

### Correlation With Clinical Scales

Given the aim of identifying clinically relevant changes in functional connectivity induced by EMDR and TF-CBT, the simple comparison of FC patterns before and after psychotherapy might be informative but also misleading. Any change in connectivity at the group level might reflect individual differences in response to therapy, as well as daily habits and other factors not related to the clinical benefit of EMDR/TF-CBT. Therefore, changes in FC were considered with respect to changes in clinical scores, i.e., CAPS, DTS, and WSAS. Separate rp-ANCOVA models were built for EMDR and TF-CBT, looking at which specific change in connectivity significantly explain changes in clinical scores.

## Results

### Demographic and Clinical Profile

**FIGURE 1:**
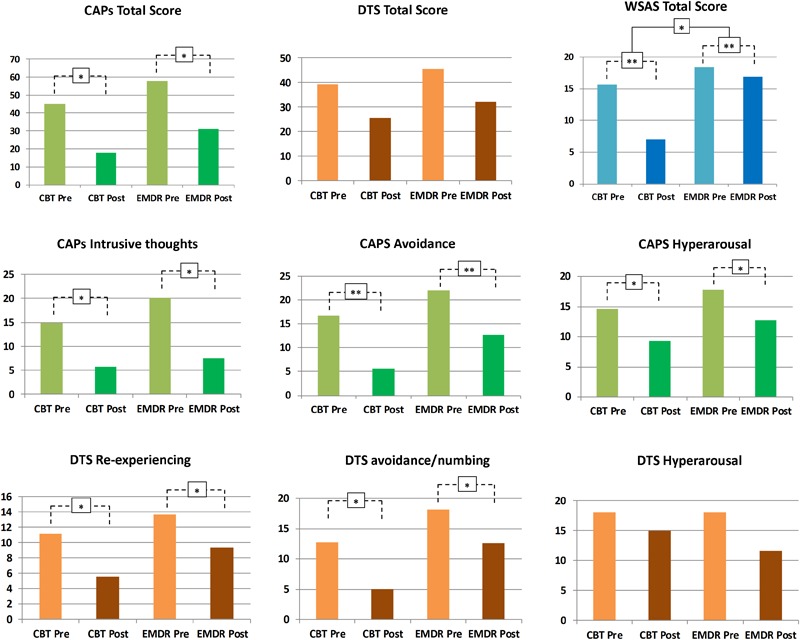
Psychotherapeutic effects on PTSD symptomatology. Changes in psychological symptoms after EMDR and TF-CBT are displayed, for both CAPS, DTS, and WSAS total scores, as well as CAPS and DTS subscales. ^∗^*p* < 0.05, ^∗∗^*p* < 0.01.

The two groups did not differ with respect to age (*t* = 0.502, *p* = 0.620), gender distribution (*X*^2^ = 0.396, *p* = 0.668) and education (*t* = 1.527, *p* = 0.140). At the time of the study a sub-sample of patients was taking psychotropic drugs (EMDR = 4, 23%; TF-CBT = 2, 14%), with no statistically significant differences among groups (*X*^2^ = 0.362, *p* = 0.639). As for medical comorbidities, two participants in the TF-CBT and three in the EMDR group reported other not-neurological/psychiatric medical conditions and were prescribed with corresponding drug therapy. Comorbidities included hypertension, diabetes and dysthyroidism. Patients were not asked to withdraw their therapy during the EMDR/TF-CBT treatment. The average scores for the different clinical scales (CAPs, DTS, and WSAS) collected at baseline evaluation in both groups are reported in Figure 1. Additional demographic and clinical information are reported in Table 1.

### Clinical Impact of EMDR/TF-CBT

#### Clinician-Administered PTSD Scale (CAPs)

As shown in Figure 1, no significant Treatment^∗^Time interaction was reported for CAPS *total* [*F*_(1,13)_ = 0.15, *p* = 0.9], with both a main effect of Time [*F*_(1,13)_ = 7.81, *p* = 0.015] and Treatment [*F*_(1,13)_ = 50.38, *p* < 0.001]. However, a marginally significant Treatment^∗^Time effect for CAPS “*intrusive thoughts*” subscale was found [*F*_(1,13)_ = 3.95, *p* = 0.068], with a marginally main effect of Time [*F*_(1,13)_ = 3.39, *p* = 0.04] and a significant main effect of Treatment [*F*_(1,13)_ = 43.14, *p* < 0.001]. CAPS *avoidance* showed no significant Treatment^∗^Time interaction [*F*_(1,13)_ = 0.1, *p* = 0.74], with significant main effect of Time [*F*_(1,13)_ = 21.94, *p* < 0.001] and Treatment [*F*_(1,13)_ = 50.17, *p* < 0.001]. CAPS *hyperarousal* showed a similar trend, with no significant Treatment^∗^Time interaction [*F*_(1,13)_ = 0.003, *p* = 0.95], a significant main effect of Treatment [*F*_(1,13)_ = 21.79, *p* < 0.001] and a marginally significant main effect of Time [*F*_(1,13)_ = 3.88, *p* = 0.04]. Overall, EMDR and TF-CBT did not show a significantly different impact on CAPS *total, intrusive thoughts, hyperarousal*, and *avoidance* scales (i.e., no significant Treatment^∗^Time interaction). Differences in the intrusive thoughts scale showed difference between EMDR and TF-CBT trending toward statistical significance, suggesting a potential greater improvement for patients in the EMDR group (see Figure 1).

#### Davidson Trauma Scale (DTS)

Total DTS score showed no significant Treatment^∗^Time interaction [*F*_(1,13)_ = 0.002, *p* = 0.96], with a significant main effect of Treatment [*F*_(1,13)_ = 7.33, *p* = 0.018] but no main effect of Time [*F*_(1,13)_ = 2.87, *p* = 0.16]. DTS *re-experiencing* showed no significant Treatment^∗^Time interaction [*F*_(1,13)_ = 0.26, *p* = 0.61], with a significant main effect of Treatment [*F*_(1,13)_ = 8.59, *p* = 0.012] and a marginally main effect of Time [*F*_(1,13)_ = 3.25, *p* = 0.04]. DTS *avoidance/numbing* showed no significant Treatment^∗^Time interaction [*F*_(1,13)_ = 0.15, *p* = 0.69], with a significant main effect of both Treatment [*F*_(1,13)_ = 7.4, *p* = 0.018] and Time [*F*_(1,13)_ = 5.55, *p* = 0.035]. Finally, DTS *hyperarousal* showed no significant Treatment^∗^Time interaction [*F*_(1,13)_ = 0.37, *p* = 0.55], with a significant main effect of Treatment [*F*_(1,13)_ = 7.19, *p* = 0.019] but no significant main effect of Time [*F*_(1,13)_ = 0.61, *p* = 0.44]. Overall, EMDR and TF-CBT did not show a significantly different impact on DTS (i.e., no significant Treatment^∗^Time interaction).

#### Work and Social Adjustment Scale (WSAS)

Total WSAS score showed a significant Treatment^∗^Time interaction [*F*_(1,13)_ = 3.36, *p* = 0.039], with both a main effect of Time [*F*_(1,13)_ = 16.56, *p* = 0.003] and Treatment [*F*_(1,13)_ = 9.44, *p* = 0.009]. EMDR and TF-CBT did exert a different impact on WSAS scores after treatment, with TF-CBT inducing greater positive changes (see Figure 1).

### Changes in FC and Predictors of Response to Psychotherapy

#### Changes in Symptomatology

**FIGURE 2:**
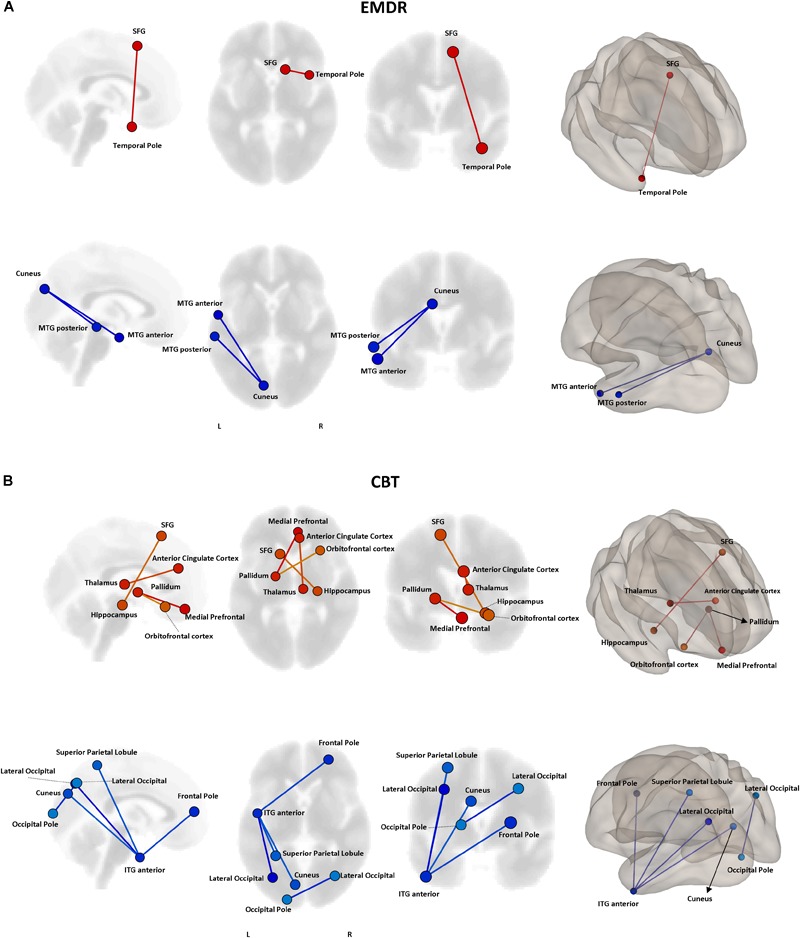
Functional connectivity and CAPS changes. Results of the repeated measures ANCOVA on pairwise connectivity and CAPS total scores are displayed for patients receiving EMDR **(A)** and TF-CBT **(B)**. Significant changes are displayed separately for increased (red) and decreased (blue) connectivity, with edges representing connections at *p* < 0.05 (FDR corrected). The strength of pre–post changes in connectivity is color-coded for both edges and nodes (yellow → red, stronger increase in connectivity; cyan → blue, stronger decrease in connectivity). Images are displayed in neurological convention. SFG, Superior Frontal Gyrus; MTG, Middle Temporal Gyrus; ITG, Inferior Temporal Gyrus; FDR, False Discovery Rate.

**FIGURE 3:**
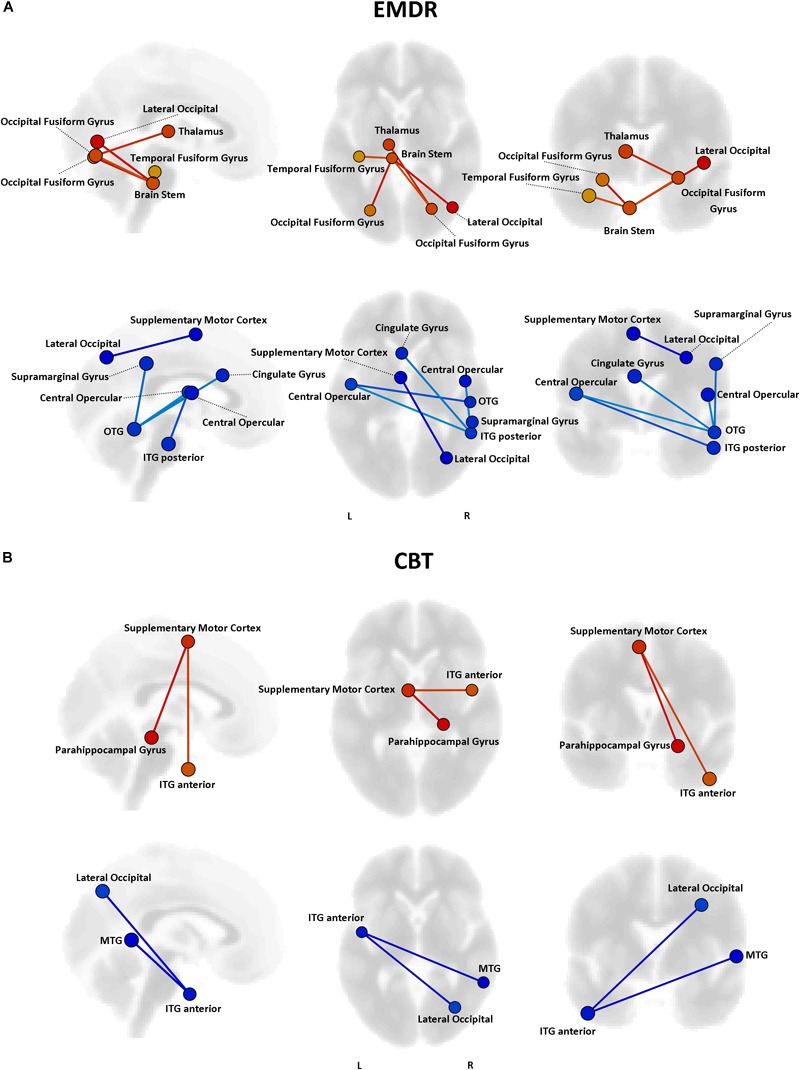
Functional connectivity and DTS changes. Results of the repeated measures ANCOVA on pairwise connectivity and DTS total scores are displayed for patients in the EMDR **(A)** and TF-CBT **(B)** groups. Significant changes are displayed separately for increased (red) and decreased (blue) connectivity, with edges representing connections with a *p* < 0.05 FDR corrected. The strength of pre–post changes in connectivity is color-coded for both edges and nodes (yellow → red, stronger increase in connectivity; cyan → blue, stronger decrease in connectivity). Images are displayed in neurological convention. MTG, Middle Temporal Gyrus; ITG, Inferior Temporal Gyrus; OTG, Occipito-Temporal Gyrus; FDR, False Discovery Rate.

**FIGURE 4:**
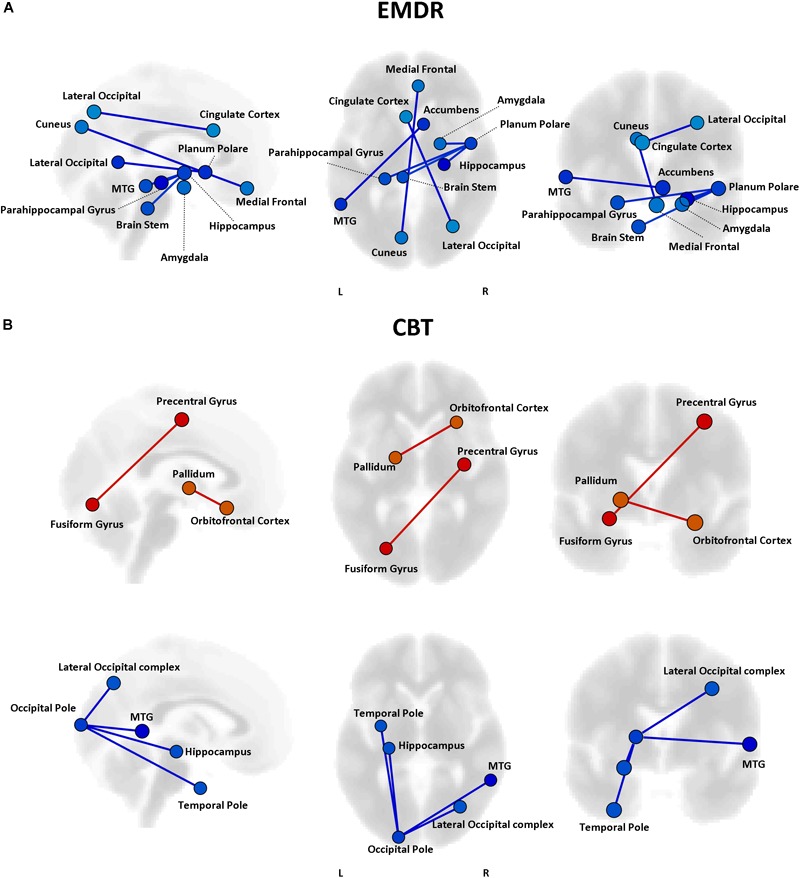
Functional connectivity and WSAS changes. Results of the repeated measures ANCOVA on pairwise connectivity and WSAS scores are displayed for patients in the EMDR **(A)** and TF-CBT **(B)** groups. Significant changes are displayed separately for increased (red) and decreased (blue) connectivity, with edges representing connections with a *p* < 0.05 FDR corrected. No increase in connectivity accompanied changes in WSAS in the EMDR group. The strength of pre–post changes in connectivity is color-coded for both edges and nodes (yellow → red, stronger increase in connectivity; cyan → blue, stronger decrease in connectivity). Images are displayed in neurological convention. MTG, Middle Temporal Gyrus; FDR, False Discovery Rate.

Even though no statistically significant differences in clinical improvement between EMDR/TF-CBT were observed (except for the WSAS), different therapy-specific rearrangements of FC could have supported the observed clinical improvement. Indeed, fMRI analysis highlighted a differential pattern of increase and decrease in connectivity possibly supporting clinical changes observed at CAPs, DTS and WSAS, for patients receiving EMDR and TF-CBT. Results for both psychotherapies and each clinical score, including subscales, are reported in Figure 2–4 and Supplementary Figure S1. Specifically, changes in pairwise FC explaining changes in CAPs score are reported in Figure 2; changes in DTS, Figure 3; changes in WSAS, Figure 4; changes in CAPs subscales (intrusive thoughts, avoidance, and hypervigilance), Supplementary Figure S1. To facilitate replication attempts, a complete list of the regions of interest included in the analyses and their corresponding MNI coordinates is reported in Supplementary Table S1.

#### Common Connectivity Changes in EMDR and TF-CBT

**FIGURE 5:**
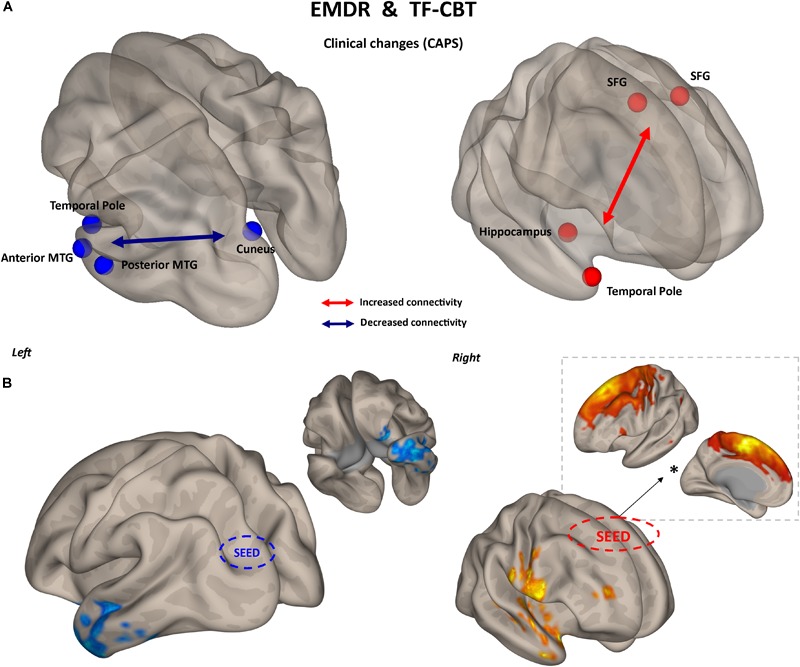
Common substrate for psychological benefit. Changes in CAPS total score are accompanied by two patterns of FC changes after both EMDR and TF-CBT **(A)**. Increased connectivity between superior frontal gyrus and right temporal pole regions, and decreased connectivity between left visual cortex and left temporal pole, explain positive changes in CAPS after psychotherapy. Results are shown for the pairwise atlas-based analysis (*p* < 0.05, FDR corrected) **(A)** and confirmed with seed-based connectivity analysis (*p* < 0.05, cluster-based correction) **(B)**. The FC profile of the seed region located in the superior frontal gyrus is also displayed (^∗^), highlighting its resemblance with the supplementary motor cortex. SFG, superior frontal gyrus; MTG, middle temporal gyrus; FDR, False Discovery Rate.

The two treatments displayed a significant heterogeneity in terms of connectivity modifications supporting changes in symptomatology. However, the analysis of overlapping regions/connections showing a similar change across the two interventions highlighted two main patterns, involving a decrease in connectivity between the left visual cortex (i.e., cuneus) and ipsilateral temporal pole [*F*_(1,29)_ = 4.76, *p* < 0.0031], as well as an increase in connectivity between bilateral superior frontal gyrus and right temporal pole structures [*F*_(1,29)_ = 4.13, *p* < 0.015] (Figure 5).

#### Connectivity-Based Predictors of Response to Therapy

**FIGURE 6:**
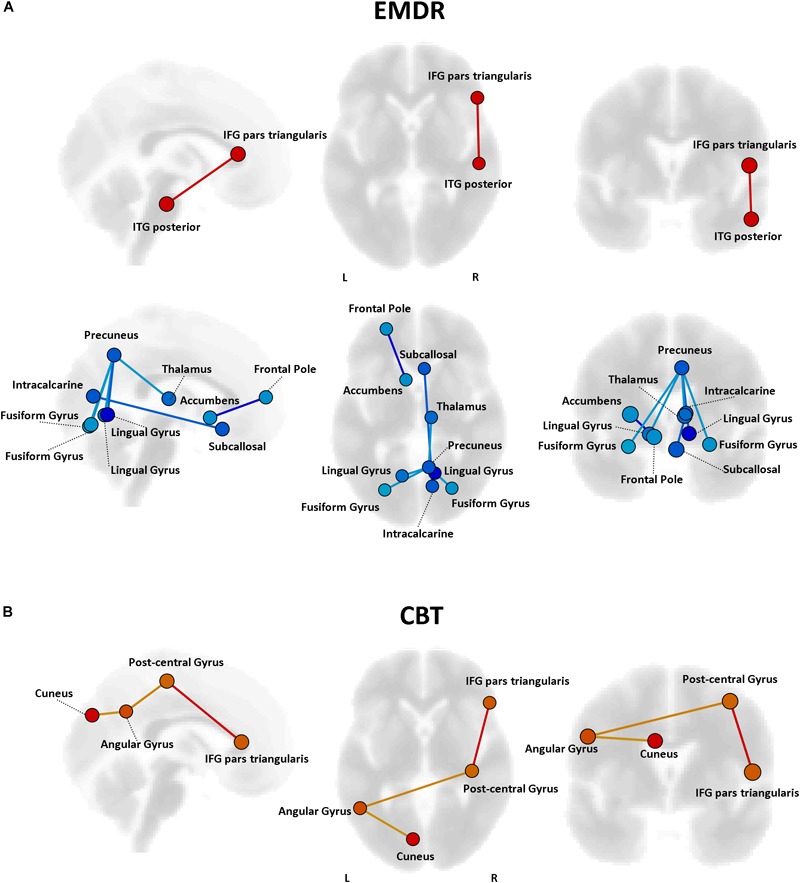
Predictors of response to psychotherapy. For both EMDR **(A)** and TF-CBT **(B)**, a set of functional connections significantly predicting changes in CAPS total score were identified (*p* < 0.05 FDR corrected). The strength of the prediction weight is color-coded (yellow → red for positive predictors; cyan → blue for negative predictors). Images are displayed in neurological convention. ITG, Inferior Temporal Gyrus; ITG, Inferior Frontal Gyrus; FDR, False Discovery Rate.

Pre-existing structural and functional brain properties of each patient might contribute to the effectiveness of any given therapy (Drysdale et al., 2016). We tested whether specific patterns of FC might predict the response to EMDR and TF-CBT, identifying different set of connections (Figure 6). Specifically, EMDR patients with decreased FC between the precuneus and visual regions seem to display a greater benefit in terms of pre–post changes at CAPS [*F*_(1,29)_ = 3.58, *p* < 0.023]. Interestingly, patients showing a benefit at CAPS (after both EMDR and TF-CBT) showed a stronger positive connectivity between the right inferior frontal gyrus (pars triangularis) and regions of the temporal lobe (for EMDR) and somatosensory cortex (for TF-CBT) [*F*_(1,29)_ = 3.49, *p* < 0.019].

## Discussion

We investigated whether two psychotherapeutic approaches, EMDR and TF-CBT, might induce significant clinical benefit in a group of PTSD patients affected by the same trauma. By using functional MRI analysis, we also measured the corresponding impact of EMDR and TF-CBT on individual FC patterns, which might possibly represent the neurophysiological substrate of psychological healing in PTSD. While both EMDR and TF-CBT exerted a beneficial effect on PTSD symptomatology, the two psychotherapeutic approaches displayed both common and dissociable effects on brain connectivity, with the overlap being represented by decreased connectivity between visual cortex and temporal lobe regions in the left hemisphere, and increased connectivity between bilateral superior frontal gyrus and right temporal pole regions.

### Psychological Impact of EMDR and TF-CBT

No significant differences were observed in the impact of EMDR and TF-CBT on PTSD symptomatology, except for a significantly greater improvement in work and social impairment following TF-CBT intervention as compared to EMDR. This is in line with existing literature, showing no evidence of greater efficacy for a specific psychotherapeutic approach in the treatment of PTSD patients (Bradley et al., 2005), especially when therapies including elements of exposure such as TF-CBT and EMDR are compared (Bradley et al., 2005). This is not surprising, considering that many treatments for PTSD share not only factors common to all psychotherapeutic approaches (Bradley and Follingstad, 2001), but also some sort of exposure therapy. Exposure fosters habituation or extinction processes, while also providing an opportunity for a controlled re-elaboration of the traumatic event, which become a core element of the therapeutic process. Similarly, therapies focused on cognitive biases or maladaptive coping strategies sometimes include element of exposure. That being said, a difference in the effectiveness of the two interventions in terms of dose-response seems present, with EMDR and TF-CBT eliciting similar results at both the clinical and neuroimaging level even though EMDR included half the number of treatment sessions (4 weekly sessions ±2) compared to TF-CBT (10 weekly sessions ±2) and an overall shorter treatment period. The present data are not suitable for a proper analysis of dose-response effects across the two approaches, but results provide an interesting insight into this matter that should be considered in future studies.

Notably, the present study offers an original evidence of the non-differential effect of EMDR and TF-CBT in PTSD patients, by providing a quantitative estimate on the same patient population and trauma-type. Conversely, this also mean that any generalization of findings to other trauma types is strongly discouraged. More in general, both interventions elicited beneficial effects on patients’ symptomatology, with a significant decrease in validated clinical scales such as CAPS and DTS. It must be noticed that, among PTSD based on different traumas, a significant variability in clinical efficacy of therapies exists, with for instance lower effect sizes for treatments of combat-related PTSD as compared to natural disaster or interpersonal violence (Ford et al., 1997), suggesting again the non-generalizability of the present results.

### Connectivity Changes Supporting Psychological Healing

The analysis of functional connectivity changes induced by EMDR and TF-CBT revealed both common and dissociable correlates for symptoms improvement recorded at the different various clinical scales. In general, both therapies seem to induce two main patterns of connectivity changes, pointing to a reduction of connectivity between regions of the visual cortex and of the left temporal pole, as well as an increase in connectivity between the superior frontal gyrus and right temporal pole. Interestingly, such changes characterize a decrease in CAPS scores in both patient groups, possibly due to the aforementioned methodological overlap between EMDR and TF-CBT for PTSD (Bradley et al., 2005).

In general, the changes in connectivity patterns highlight the involvement of the bilateral temporal pole. Changes in these structures have been extensively documented in PTSD patients (Shin, 2006; Cheng et al., 2015; Meng et al., 2016), including recent results about changes in hippocampal volume induced by EMDR treatment (Bossini et al., 2017). The specific decrease in connectivity between regions of the occipital cortex (e.g., cuneus) and the left temporal pole might point to a reduction of spontaneous synchronization between visual processing areas and re-elaboration of traumatic events (including flashbacks) which might be prompted by temporal lobe structures (Kroes et al., 2011). Interestingly, this correlation also appears to specifically characterize the intrusive thoughts subscale of CAPS, but not the avoidance and hyper-arousal ones. Models of (visual) flashbacks generation suggest a dominance of the activity in the dorsal visual stream, which includes posterior visual to superior parietal regions (including the cuneus and precuneus) and is responsible for processing of egocentric (i.e., own viewpoint) representations of experience. While the dorsal visual stream elaborates trauma-related representations associated with the insula and amygdala (reflecting emotional and body state responses), the ventral visual stream, including inferior and middle temporal regions, enables scenes to be visualized allocentrically (i.e., from alternative viewpoints), and provides memories with their context (Brewin et al., 2010). The observed therapy-related changes might suggest a modification of the ventral-dorsal stream balance.

An increase in connectivity between regions of the prefrontal cortex (i.e., superior frontal gyrus) and right temporal pole fits with the general neurocognitive theory about the beneficial effect of psychotherapy, which postulate an increase in top-down control as the main mechanism behind psychological healing in (among others) anxiety, trauma-related and addiction disorders (Robertson et al., 2004; Malejko et al., 2017). For instance, in PTSD in particular, impaired top-down cognitive control over limbic areas, which is frequently associated with hypo-activation in the dorsolateral prefrontal cortex, has been linked to the persistence of traumatic flashbacks as well as to worsening of attention (White et al., 2015). Increased connectivity between prefrontal and temporal pole regions might reflect a greater control of trauma-related contents, decreasing their intrusiveness during spontaneous mind wandering (Kroes et al., 2011). This also matches recent finding of resting-state fMRI networks alterations in PTSD patients with the same trauma-type as those enrolled in the present study (i.e., earthquake) (Shang et al., 2014). At a very general level, the authors reported modification of FC in various brain networks including the salience network (SN), central executive network (CEN), default mode network (DMN), somato-motor network (SMN), auditory network (AN), and visual network (VN). Differently from networks related to primary sensory systems (i.e., visual, auditory, and motor), activity in, e.g., DMN, SN, and CEN is associated with higher order cognitive dynamics, more specifically related to executive functioning (CEN), memory (CEN, DMN), attention (SN, CEN), monitoring of bodily sensation (SN) and mind wandering (DMN) (for a review see Zhang and Raichle, 2010). In general, this suggest changes in PTSD not being confined to sensorial processing, but also possibly involving cognitive networks. Interestingly, Shang and colleagues also observed that stronger connectivity involving the inferior temporal gyrus (ITG) and supplementary motor area (SMA) was negatively correlated with clinical severity in PTSD patients. The location of the superior frontal gyrus in our atlas highly resemble SMA (see Figure 5), while the ITG is one of the multiple temporal lobe regions showing increased connectivity with SMA after psychotherapy in our sample. This might be suggesting that both EMDR and TF-CBT work by re-normalizing such altered SFG/SMA ←→ temporal gyrus connectivity, confirming the potential pivotal role of this specific functional connection in PTSD patients’ symptomatology.

The analysis of predictors of response to therapy highlighted different connectivity patterns for EMDR and TF-CBT, with some overlap for the inferior frontal gyrus, and higher predictive power for regions previously highlighted in relation to the response to therapy, e.g., the cuneus. Moreover, a role for decreased connectivity of the precuneus was also identified. It is important to note that all the potential predictors identified in the present analysis require a careful validation via ad-hoc studies investigating their correlation with cognitive and clinical scores, and are here discussed as additional exploratory findings. The finding about increased cuneus connectivity at baseline fits with the reduction in connectivity observed after therapy, suggesting that patients with higher connectivity of the visual cortex before therapy are possibly those observing a greater response to EMDR/TF-CBT. As for the precuneus –a crucial node of the DMN— multiple studies have pointed out alterations of precuneus connectivity (and of the DMN in general) in PTSD patients (Boccia et al., 2016). During memory retrieval –a crucial component for flashbacks generation— images are manipulated in terms of their content and point of view. Such conversion between egocentric and allocentric reference frames is assumed to be supported by the retrosplenial and posterior parietal cortices, with imagery supported instead by the precuneus. Decreased connectivity between precuneus and areas of the visual cortex might point to the aforementioned ventral-dorsal stream framework, with a decrease in integration between precuneus and visual areas suggesting a less efficient shift from ego- to allo-centric images in patients before therapy. Finally, the IFG might be relevant for its role in inhibition processes, whose alterations have been reported in several studies on PTSD. For instance, decreased IFG activation during a proactive inhibition task in combat veterans as compared with a combat control group have been reported (van Rooij et al., 2014), while increased IFG resting-state fMRI activity has been recently suggested in a quantitative meta-analysis of fMRI findings in PTSD patients (Wang et al., 2016).

### Insight for Further Combined Therapeutic Approaches

Non-invasive brain stimulation (NIBS), and transcranial electrical stimulation (tES) in particular, are becoming pivotal tools for the investigations of neuromodulatory intervention in both the healthy and pathological brain (Filmer et al., 2014; Bestmann et al., 2015; Santarnecchi et al., 2015). The possibility of applying low voltage electrical stimulation patterns to modulate –excite or inhibit— the activity of specific brain regions or entire networks constitutes an appealing scenario (e.g., using transcranial Direct Current Stimulation, tDCS) (Nitsche and Paulus, 2011), with potential applications for both the causal investigation of brain-function dualism [following the “virtual-lesion” approach (Pascual-Leone and Pridmore, 1995; Pascual-Leone et al., 1999)], as well as for the enhancement of individual cognitive functioning (Polania et al., 2012; Sela et al., 2012; Santarnecchi et al., 2013, 2016; Snowball et al., 2013). Additionally, recently developed techniques such as transcranial alternating current (tACS) and transcranial random noise (tRNS) stimulation offer the possibility to modulate brain activity by interacting with cortical excitability and/or specific brain oscillatory dynamics as those recorded via electroencephalography (EEG), exponentially multiplying potential available interventions (Thut et al., 2012). In this framework, with the increasing spatial resolution of current tES modeling works (Datta et al., 2009) and the potential to indirectly stimulate subcortical structure using Transcranial Magnetic Stimulation (TMS) (Wang et al., 2014), NIBS is becoming a valuable tool for the treatment of both neurological and psychiatric conditions, with FDA-approved protocols already available for conditions such as Depression and Obsessive Compulsive Disorder (Pascual-Leone et al., 1996). The present results, together with previously reported findings in PTSD patients, might suggest potential targets for both TMS and tES applications aimed at enhancing the therapeutic processes induced by psychotherapy. For instance, application of cathodal tDCS over the occipital lobe in PTSD patients might decrease local cortical excitability and modulate connectivity patterns (Callan et al., 2016; Hauser et al., 2016), and could be used to amplify the effect of each therapeutic session. Following the same logic, increase in excitability of prefrontal regions could be achieved by means of anodal tDCS, possibly increasing top-down control over subcortical regions. Given appropriate neurophysiological investigations aimed at defining the target EEG frequency band, a de-synchronization of occipital and temporal lobes activity in the left hemisphere could be hypothesized by applying tACS with opposite stimulation phase on the two lobes (i.e., 180^∗^ phase, “anti-phase”). Solutions targeting resting-state, large scale networks including the aforementioned target regions could also constitute valuable therapeutic solutions (Ruffini et al., 2018). Studies combining EEG and fMRI recording in patients before and after psychotherapy are needed to carefully defined stimulation patterns.

### Limitations of the Study and Future Directions

Future investigations should include a placebo and/or wait-list control condition, and also compare EMDR and TF-CBT with other available approaches such as mindfulness-based therapies (King et al., 2016a), especially given the specific functional and structural effects of mindfulness practice on the brain (Holzel et al., 2011; Santarnecchi et al., 2014). The same comparison should also be explored in PTSD patients with different traumatic events.

Moreover, it should be noticed that, for different clinical scales, patients in both groups did show changes in connectivity of the thalamus (EMDR for DTS, TF-CBT for CAPs). Prior investigations using functional imaging have showed evidence of thalamic dysfunction in PTSD patients (e.g., Lanius et al., 2001; Francati et al., 2007). Future studies should look into the specific effects of psychotherapy on PTSD patients’ thalamic function, with a finer characterization of FC patterns of different thalamic nuclei, and also including perfusion imaging data (arterial spin labeling – ASL).

Finally, the present investigation is based on a pseudo-randomized assignment to EMDR and TF-CBT across patients based on patients’ trauma severity at presentation. While this might represent a reasonable solution to ensure a balanced comparison of treatment effects in a relatively small pilot study such as the present one, future investigation should adopt a fully randomized assignment in larger samples of PTSD patients.

## Conclusion

Results point to a similar, beneficial psychological impact of EMDR and TF-CBT psychotherapeutic interventions for treatment of natural disaster-related PTSD patients. Also, fMRI data suggest a similar neurophysiological substrate for the observed clinical improvement following EMDR and TF-CBT, involving connectivity changes affecting bilateral temporal pole structures. This might point to the presence of a general psychological and neurophysiological effect of exposure- and reprocessing-based psychotherapy for natural-disaster PTSD, with a minor role played by therapy-specific components.

## Author Contributions

ES designed the study, acquired MRI data, analyzed the MRI data, and wrote the manuscript. LB designed the study and conducted the psychiatric assessment. GV acquired the MRI data. PLP coordinated the EMDR and TF-CBT sessions. GDL interpreted the results. AF, AS, SR, and AR interpreted the results and edited the manuscript.

## Conflict of Interest Statement

The authors declare that the research was conducted in the absence of any commercial or financial relationships that could be construed as a potential conflict of interest.

## Footnotes

1 www.fil.ion.ucl.ac.uk/spm/
