# Supplementary material for: Psychological and Brain Connectivity Changes Following Trauma-Focused CBT and EMDR Treatment in Single-Episode PTSD Patients
Source: Front Psychol. 2019 Feb 25;10:129. doi: 10.3389/fpsyg.2019.00129 (PMC6397860; doi:10.3389/fpsyg.2019.00129)
Supplement: Supplementary file 1 [file Data_Sheet_1.docx]

**Psychological and Brain Connectivity Changes Following Trauma-Focused CBT and EMDR Treatment in Single-Episode PTSD Patients**

Emiliano Santarnecchi^1-2^, Letizia Bossini^3^, Giampaolo Vatti^1^, Andrea Fagiolini^3^, Patrizia La Porta^3^, Giorgio Di Lorenzo^4^, Alberto Siracusano^4^, Simone Rossi^1^, Alessandro Rossi^5^

^1^ Siena Brain Investigation & Neuromodulation Lab, Department of Medicine, Surgery and Neuroscience, Neurology and Clinical Neurophysiology Section, University of Siena, Italy

^2^ Berenson-Allen Center for Non-Invasive Brain Stimulation, Beth Israel Deaconess Medical Center, Harvard Medical School, Boston, MA, USA

^3^ Psychiatry Department, University of Siena, Siena, Italy

^4^ Psychiatry and Clinical Psychology Unit, Department of Neurosciences, Fondazione Policlinico “Tor Vergata”, Rome, Italy

^5^ Department of Medicine, Surgery and Neuroscience, University of Siena School of Medicine, Siena, Italy

**Table S1. Regions of interest for connectivity analysis.** The cortical and subcortical anatomical regions included in the fMRI analysis are listed with their corresponding spatial coordinates in MNI space. Note: L=left; R=right; ant=anterior; post=posterior; sup=superior; inf=inferior; MNI= Montreal Neurological Institute.

**
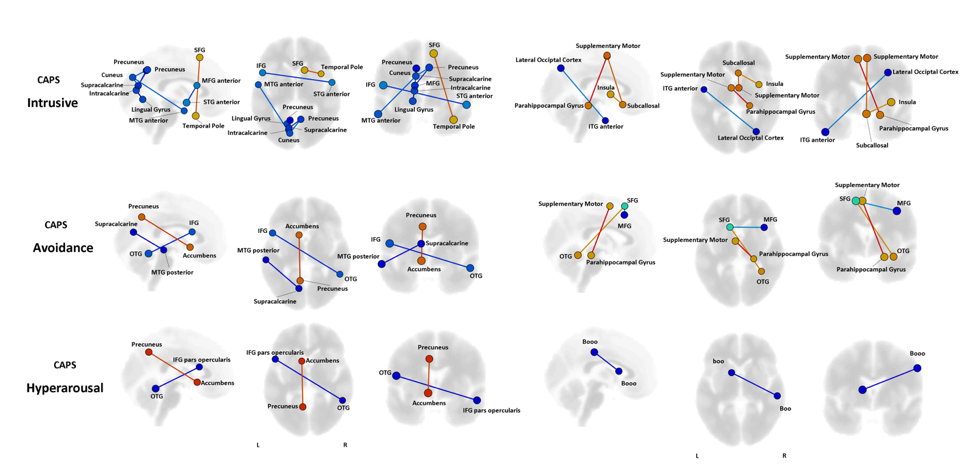
**

**Figure S1. Functional connectivity and CAPS subscales.** Results of the repeated measures ANCOVA on pairwise connectivity and changes in each subscale of the CAPS are displayed for patients in the EMDR and TF-CBT groups. Significant increased (red) and decreased (blue) edges represent connections with a p.<0.05 FDR corrected. Images are displayed in neurological convention. Note: SFG=Superior Frontal Gyrus; MTG=Middle Temporal Gyrus; ITG= Inferior Temporal Gyrus; Inferior Frontal Gyrus; Middle Temporal Gyrus; Superior Temporal Gyrus; FDR=False Discovery Rate**.**
